# The Anatomic Course of the First Jejunal Branch of the Superior Mesenteric Vein in Relation to the Superior Mesenteric Artery

**DOI:** 10.1155/2012/538769

**Published:** 2012-02-22

**Authors:** Pavlos Papavasiliou, Rodrigo Arrangoiz, Fang Zhu, Yun Shin Chun, Kristin Edwards, John P. Hoffman

**Affiliations:** ^1^Department of Surgical Oncology, Fox Chase Cancer Center, Philadelphia, PA 19111, USA; ^2^Department of Biostatistics, Fox Chase Cancer Center, Philadelphia, PA 19111, USA; ^3^Department of Radiology, Fox Chase Cancer Center, Philadelphia, PA 19111, USA

## Abstract

*Introduction*. The purpose of this study is to determine the anatomic course of the first jejunal branch of the superior mesenteric vein (SMV) in relation to the superior mesenteric artery (SMA). *Methods*. Three hundred consecutive contrast-enhanced computed tomography (CT) scans were reviewed by a surgical oncologist with confirmation of findings by a radiologist. *Results*. The overall incidence of a first jejunal branch coursing anterior to the SMA was 41%. There was no correlation between patient gender and position of the jejunal branch. In addition, there was no correlation between size of the first jejunal branch and its location in relation to the SMA. The IMV drained into the SMV in 27% of the patients. The IMV drained into the SMV-portal vein confluence in 17% of patients and inserted into the splenic vein in 54%. An anterior coursing first jejunal branch statistically correlated with an IMV that drained into the SMV-portal vein confluence (*P* = 0.009). *Conclusion*. The first jejunal branch of the SMV has a highly variable course in relation to the SMA and has a higher incidence of an anterior location in this population than previously reported.

## 1. Introduction

Pancreatoduodenectomy offers many technical challenges in the treatment of pancreatic cancer. Recent studies have shown a benefit to neoadjuvant treatment for pancreatic cancer with vascular involvement [1–3]. This has led to more extensive and complex pancreatic resections with venous reconstruction [4]. The complexity of these procedures places an even higher importance on extensive preoperative knowledge of mesenteric venous anatomy, its relationship to arterial anatomy, and the improved quality of preoperative computed tomography (CT) gives the surgeon a tool to “map out” planned resections and reconstructions.

The first-order branches of the superior mesenteric vein (SMV) are the ileal and jejunal branches. The first jejunal branch has variable anatomy with relation to the superior mesenteric artery (SMA) as it courses either anterior or posterior to the artery. Venous drainage of the uncinate process of the pancreas to the SMV, jejunal, and ileal branches varies according to the anatomic course of these branches with relation to the SMA. In order to adequately expose the SMA for a clear margin of resection, these small venous branches to the SMV and its branches must be carefully ligated. Posteriorly located branches receive most of the drainage of the uncinate, while patients with an anteriorly located branch drain through the ileal branch, making SMA exposure less difficult [5]. If a surgeon is unaware of the position of this branch relative to the SMA, serious injury and massive blood loss may occur. The primary objective of this study was to determine the distribution of anatomic variations of the first jejunal branch of the SMV in relation to the SMA in a western population using CT imaging that would routinely be used by surgeons in the preoperative setting.

## 2. Methods

Three hundred consecutive computed tomographic (CT) scans with intravenous contrast of the abdomen and pelvis performed between August 1, 2010 and August 23, 2010 were reviewed by a surgical oncologist with confirmation of findings by a radiologist. Institutional review board approval was obtained prior to the study for review of CT scans. All CT scans were performed using a SOMATOM Sensation 64 (Siemens, Munich, Germany), with continuous 5 mm thick sections and 5 mm intervals. Reconstruction parameters were set at a 1.5 mm thickness and 0.8 mm intervals. Injection of 80 mL of iopamidol (Isovue 370, Bracco diagnostics, Princeton, NJ, USA) at a rate of 2 mL per second was performed via a 20-gauge intravenous catheter, followed by cross-sectional imaging of the abdomen and pelvis 70 seconds after injection.

Data recorded included gender of the patient, the relationship of the first jejunal branch of the SMV to the SMA, and diameter of the jejunal and ileal branches, whether the jejunal and ileal branches formed a common trunk prior to joining the splenic vein and drainage of the inferior mesenteric vein (IMV). If a consensus on the anatomy was not reached between the two readers, the radiologist's interpretation was used in the analysis.

**Figure 1:**
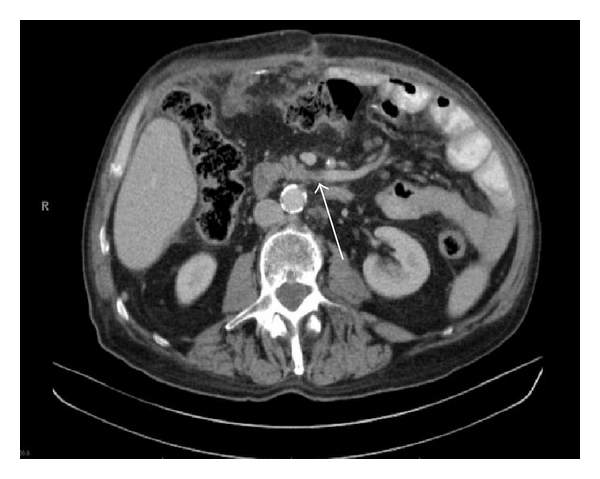
Posterior coursing first jejunal branch.

**Figure 2:**
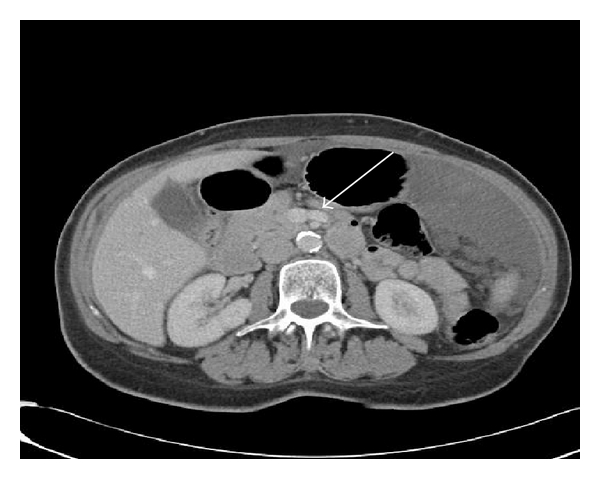
Anterior coursing first jejunal branch.

The first jejunal branch was defined as the most caudal branch draining proximal jejunal loops into the SMV identified on axial images. The first jejunal branch was classified as either posterior or anterior depending on its position with relation to the SMA. Vein diameter was recorded for the jejunal and ileal branches immediately prior to forming the SMV on axial imaging (Figures 1 and 2).

The IMV was identified by tracing the venous drainage from the sigmoid mesocolon along the left paraspinal area, anterior to the left renal vein, then into the splenic vein, splenoportal vein confluence, SMV, or SMV branches. The IMV was classified based on which of these veins it drained into.

Statistical analysis included frequency, proportion and the 95% confidence interval of an anterior, and posterior first jejunal branch of the SMV in relation to the SMA. Correlation between gender of the patient, diameter of the first jejunal branch, IMV drainage, and the presence of a common SMV trunk was determined with anatomic course of the first jejunal branch of the SMV. All tests that were two-sided with a *P* value less than 0.05 considered significant.

With 300 patients, the width of the 95% CI for the proportion estimate of anterior (or posterior) jejunal branch was 0.06 when the true proportion is 50%, and we were able to detect a difference of 8% from 50% using a binomial test with 80% power.

## 3. Results

Gender distribution included 137 (46%) men and 163 (54%) women. The anatomic course of the first jejunal branch was identifiable in all CT scans reviewed using axial images, and coronal images were used if clarification of anatomy was needed (Table 1).

The overall incidence of an anterior coursing first jejunal branch was 41%. Sixty (44%) men had an anterior coursing branch and 63 (39%) women had an anterior coursing branch. There was no correlation between patient gender and course of the first jejunal branch (*P* = 0.39). In 16 (5%) patients, the jejunal and ileal branches drained together into the splenoportal confluence.

Mean overall jejunal branch diameter was 7.9 mm compared to 10.1 mm for the ileal branch. Mean vein diameter for anterior branches was 7.9 mm compared to 7.6 mm for posterior branches. There was no correlation between jejunal or ileal branch diameter and anatomic course, although an anterior coursing first jejunal branch had a trend toward larger diameter (*P* = 0.07) (Table 2).

The IMV drained into the splenic vein in 163 (54%) patients, into the splenoportal vein confluence in 52 (17%) patients, and into the SMV trunk in 82 (27%) patients. The IMV drained into an anterior first jejunal branch in two (0.67%) patients and into the ileal branch in one (0.3%) patient. An IMV that drained into the splenoportal vein confluence significantly correlated with an anterior coursing first jejunal branch (*P* = 0.009).

**Figure 3:**
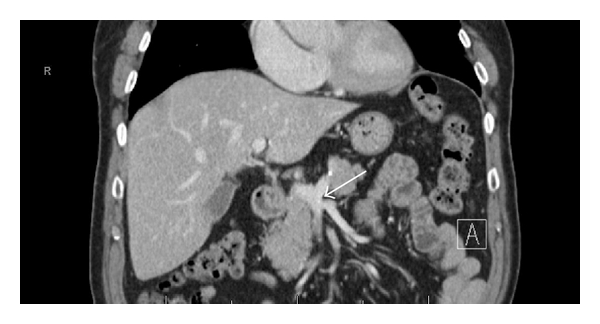
No Common SMV Trunk. Jejunal and ileal branches joining at splenoportal confluence.

Sixteen patients (5.3%) did not have a common SMV trunk, with both ileal and jejunal branches draining together into the splenoportal confluence. All patients without a common SMV trunk had an anterior coursing first jejunal branch (Figure 3).

## 4. Discussion

The variability in mesenteric venous anatomy poses a challenge for surgeons during pancreatoduodenectomy, especially with borderline resectable pancreatic cancers [6], where venous resection and reconstruction may be required to obtain a negative margin. Tseng et al. reported on 110 patients diagnosed with adenocarcinoma of the pancreas that required vascular resection and reconstruction. No difference in overall survival was observed when these patients were compared to 181 other patients who did not undergo vascular resection (23.4 months versus 26.5 months, *P* = 0.18) [7]. Yebekas et al. reported similar results in 2008, demonstrating that patients who underwent vascular resection had no difference in overall survival whether or not there was evidence of vascular invasion on final pathology [8]. In fact, both studies reported that node positive disease was the strongest predictor of outcome in these patients. These studies demonstrate the importance of vascular resection in select patients with pancreatic adenocarcinoma, and preoperative knowledge of each patient's anatomy is crucial is planning for resection and reconstruction. The ability to identify a patient's mesenteric venous anatomy with preoperative CT scans can also avoid major blood loss by anticipating the course of the veins. Avoiding injury to these branches also prevents injury to the SMA that can occur in an attempt to control venous bleeding with sutures.

There have been several studies evaluating mesenteric anatomy using CT scans [9–13]. However, to our knowledge, only two studies from Eastern populations have specifically examined anatomic variants of the first jejunal branch. Both studies were conducted with the purpose of classifying mesenteric venous anatomy using noninvasive imaging, as there were very few reports describing the anatomy of the SMV in the literature.

Kim et al. retrospectively reviewed 220 patients who had CT scans with venous phase IV contrast, and defined the first jejunal branch as the first venous branch draining proximal jejunal loops. An anterior coursing branch was identified in 19% of patients [14]. Sakaguchi et al. used 3-dimensional portography to classify anatomic variants of mesenteric veins in 107 consecutive patients. The incidence of an anterior first jejunal branch or trunk was 32.4% in that population [15].

This study found an overall incidence of an anterior coursing first jejunal branch of 41%, which is significantly higher than the rates mentioned in the above two studies. Mesenteric venous anatomy is not only variable between individuals, but also between populations which places an even greater importance on identification and understanding of this anatomy preoperatively. However, this is the largest study known to the authors to specifically characterize the course of the first jejunal branch of the SMV using conventional CT imaging that most surgeons use in the preoperative setting.

The IMV drained into the SMV in 27% of our patients, with one patient draining into the ileal branch and two into the jejunal branch. Preoperative CT imaging gives surgeons an opportunity to trace these tributaries from their drainage source so that an IMV draining into the SMV is not mistaken for a first jejunal branch intraoperatively. Our IMV drainage distribution rates are similar to other anatomic studies that examined this [11, 14, 15].

Statistical correlation was found between an anterior coursing first jejunal branch and an IMV draining into the SMV-portal vein confluence. In addition, all patients without a common SMV trunk had an anterior coursing first jejunal branch. Patients without a common SMV trunk could pose a challenge if venous reconstruction is required for resection, and preoperative knowledge of this anatomy allows development of an optimal plan for reconstruction.

No statistical significant difference between anterior and posterior first jejunal branch diameter was found, although there was a trend for anterior branches to be larger. This may indicate that anterior branches harbor more of the intestinal venous drainage than posterior branches, and that anterior branches may be more amendable to reconstruction.

Mean overall ileal branch diameter was larger than mean overall jejunal branch diameter, illustrating why ileal branch reconstruction is usually preferred over jejunal branch reconstruction [16].

The anatomic course of the first jejunal branch of the SMV is highly variable but readily identifiable using standard CT imaging with IV contrast. Preoperative identification of the course of the first jejunal branch, in addition to drainage patterns of the IMV can aid surgeons in planning for pancreatoduodenectomy with or without venous reconstruction.

## Figures and Tables

**Table 1:** Anatomic distribution.

	Number (percentage)
Gender	
Male	137 (46%)
Female	163 (54%)
1st Jejunal branch course	
Anterior to SMA	123 (41%)
Posterior to SMA	177 (59%)
No SMV trunk	16 (5%)
IMV drainage	
Splenic vein	163 (54%)
Common trunk	52 (17%)
SMV	82 (27%)
Ileal branch	1 (0.3%)
Jejunal branch	2 (0.67%)

**Table 2:** Mean vein diameter.

Jejunal branch	7.8 mm
Anterior jejunal	7.9 mm
Posterior jejunal	7.7 mm
Ileal branch	10.2 mm
